# Using Typed Dependencies to Study and Recognise Conceptualisation Zones in Biomedical Literature

**DOI:** 10.1371/journal.pone.0079570

**Published:** 2013-11-18

**Authors:** Tudor Groza

**Affiliations:** School of Information, Telecommunications, and Electronics Engineering, The University of Queensland, Queensland, Australia; University of Illinois-Chicago, United States of America

## Abstract

In the biomedical domain, authors publish their experiments and findings using a quasi-standard coarse-grained discourse structure, which starts with an introduction that sets up the motivation, continues with a description of the materials and methods, and concludes with results and discussions. Over the course of the years, there has been a fair amount of research done in the area of scientific discourse analysis, with a focus on performing automatic recognition of scientific artefacts/conceptualisation zones from the raw content of scientific publications. Most of the existing approaches use Machine Learning techniques to perform classification based on features that rely on the shallow structure of the sentence tokens, or sentences as a whole, in addition to corpus-driven statistics. In this article, we investigate the role carried by the deep (dependency) structure of the sentences in describing their rhetorical nature. Using association rule mining techniques, we study the presence of dependency structure patterns in the context of a given rhetorical type, the use of these patterns in exploring differences in structure between the rhetorical types, and their ability to discriminate between the different rhetorical types. Our final goal is to provide a series of insights that can be used to complement existing classification approaches. Experimental results show that, in particular in the context of a fine-grained multi-class classification context, the association rules emerged from the dependency structure are not able to produce uniform classification results. However, they can be used to derive discriminative pair-wise classification mechanisms, in particular for some of the most ambiguous types.

## Introduction

In the biomedical domain, authors publish their experiments and findings using a quasi-standard coarse-grained discourse structure. This starts with an introduction that sets up the background and motivation, continues with a description of the materials and methods, and concludes with results and discussions. At a lower granularity level, we find scientific artefacts, or conceptualisation zones, for example, hypotheses: “*Since the dipole moment change induced by the puckering vibration will be perpendicular to the ring plane and along the c-inertial axis, the vibrationrotation transitions are expected to follow c-type selection rules.*” (as per the ART corpus [1]) or findings: “*The binding of both forms of 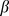-catenin to CBP is completely inhibited by ICG-001*” (as per the Shatkay corpus [2]). These provide a rhetorical structure to knowledge. As pointed by Liakata et al. in [1], the analysis of this fine-grained discourse structure enables us to differentiate between the nature of the knowledge captured in the elementary discourse units and domain-specific events. Furthermore, modelling and acquiring the explicit types externalised by this discourse analysis improves the distinction between established facts and speculative statements or between existing and new work. Finally, it provides the scaffolding required to create knowledge networks, i.e., graphs of linked statements that span across multiple publications.

There has been a fair amount of research done in the area of scientific discourse analysis, with a focus on creating ontological models for the rhetorical structure or on performing automatic recognition of scientific artefacts or conceptualisation zones from the raw content of biomedical publications. In the first category, we find a varied series of rhetorical and argumentation schemes, some of which capture only the coarse-grained structure, e.g., [3]–[5], others concentrate on the relations between fine-grained discourse elements, e.g., [6], while others combine larger rhetorical blocks with elementary units and their associated relations, e.g., [7]–[9]. In addition to these, there is an entire field that discusses and analyses pure argumentation models. The second category can be split into: (i) sentence or zone recognition or classification, according to a predefined annotation scheme [1], [10]–[12], and (ii) detection and analysis of speculative language and hedging [13]–[15]. In addition to the above, we can mention the work of Shatkay et al. [2] (initiated by Wilbur in [16]), who aimed to perform classification using a multi-dimensional scheme, and hence covered to some extent both directions – i.e., focus not only the rhetorical type of the sentence, but also the degree of speculation or the experimental evidence.

In the context of this article, we are interested in the second category, and more specifically, in the sentence-based classification. All existing classification approaches have a shared generic experimental structure: (i) they all use Machine Learning techniques to perform classification – e.g., Naive Bayes, Conditional Random Fields [17] or Support Vector Machines [18]; and (ii) the features used for classification rely on the shallow structure of the sentence tokens, or sentences as a whole, in addition to corpus-driven statistics. Among the most widely used features are: Part-Of-Speech (POS) tags, verbs classes, n-gram models of the tokens often encountered in the corpus, positioning within the document structure or the title of the corresponding section within the publication. Document structure-based features are missing, for example, in Shatkay and Wilbur's experiments [2] as their corpus is built from detached randomly chosen sentences from random publications. The experimental results reported so far (e.g., 42% average F1 score across 11 classes achieved Liakata et al. [1] – F1 ranges between 18% and 76%) are very promising, and this encourages us to continue working on the topic.

In this article, we aim to advance the state of the art by investigating the role carried by the deep (dependency) structure of the sentences in describing their rhetorical nature. A dependency structure represents a directed graph between the tokens of a sentence, where edges denote pairwise grammatical relationships, e.g., *determiner* (the – man) or *adverbial modifier* (genetically – modified). Here, we study:

- the presence of dependency structure patterns in the context of a given rhetorical types.
- the use of these patterns in exploring the differences in structure between the rhetorical types, within and across different corpora, and
- the ability of these patterns to discriminate between the different rhetorical types.

Our final goal is to provide a series of insights that can be used to complement existing classification approaches.

In order to achieve our goals we employ association rule mining on the dependency structure of the sentences. This results in a set of association rules, which encode patterns between the nodes, i.e., dependency relations, of the dependency structure. Subsequently, these association rules can be used for classification, i.e., studying their discriminative power, and more importantly, to explore the differences in structure between the rhetorical types. As opposed to some of the standard Machine Learning features used so far for classification, e.g., n-grams derived from corpus analysis, rules are domain-agnostic, as they use strictly the local linguistic perspective of the sentence. Moreover, they provide a clear and transparent method to compare sentences, independently of the rhetorical type or corpus. This last aspect is particularly important as it enables cross-corpus analysis, mapping and even classification, independently of the underlying annotation scheme or granularity.

In practice, this article describes the experiments and lessons learned from applying the above mentioned techniques on two of the existing corpora used for recognising conceptualisation zones. The two corpora are: (i) the ART corpus [1], [19], [20], which focuses on biochemistry, and (ii) the corpus developed by Shatkay et al. [2], which has no domain focus (from hereon we will refer to this corpus as the Wilbur corpus). We will show later in the article that, except for the generic goal, they differ in all other aspects, including the annotation scheme, granularity of rhetorical types and annotations and target domain, which increases the complexity of the tasks associated with our goals.

## Materials and Methods

**Figure 1:**
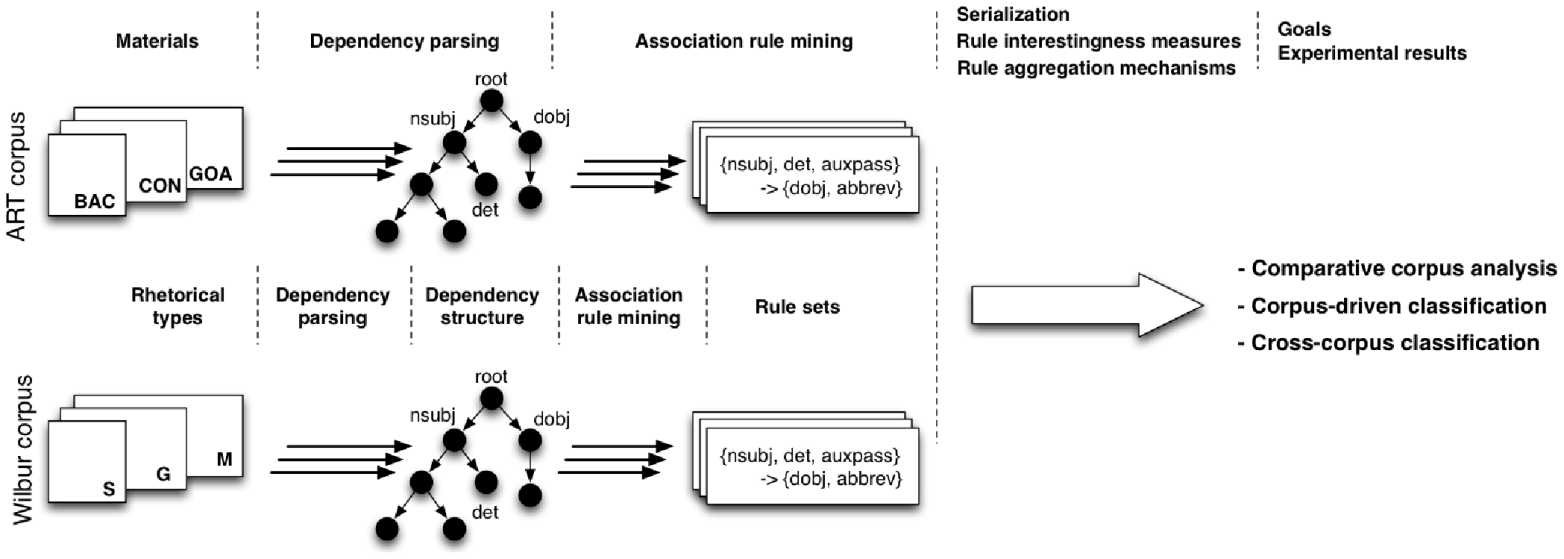
Methodology and approach roadmap. The diagram depicts the steps performed by our approach and (in the upper part) the sections in the paper where these steps are described. We start by discussing the corpora used within our experiments, then describe the dependency parsing process which results in dependency structures associated to each sentence in the corpora. Association rule mining is employed on the dependency structures and the final rule sets are filtered and aggregated using diverse serialisations, rule interestingness measures and aggregation mechanisms. These steps enable us to qualitatively compare the two corpora, and to perform corpus-driven and cross-corpus classification.

In this section we describe the data and methods used in our experiments. We start with a description of the two corpora, ART and Wilbur, then provide a brief background on dependency parsing and finally, we introduce association rule mining and the aggregation mechanisms we have applied for classification. Fig. 1 depicts a high-level overview of the methodology and can be used as a roadmap both for our approach as well as for the content of this paper.

### Ethics Statement

N/A.

### Materials

We performed experiments using two openly available corpora, previously used for automatic recognition of conceptualisation zones: the ART corpus [1], [9], [20] and the multi-dimensional classification corpus compiled by Shatkay et al. [2].

The ART corpus comprises a set of 265 scientific publications (39,915 sentences) from physical chemistry and biochemistry. Each publication has been manually annotated at sentence level by at least three annotators using the Core Scientific Concepts (CoreSC) scheme [8], [20]. The CoreSC annotation scheme defines 11 rhetorical categories, some of which may have novelty and polarity attributes associated. The novelty factor (annotated via New or Old) shows whether the statement/method is attributed to the current paper or to a previously published one, while polarity (denoted via Advantage or Disadvantage) provides a positive or negative interpretation of the author over a method previously proposed. The categories defined by the scheme (and their coverage in the corpus) are: Hypothesis (HYP –2%), Motivation (MOT –1%), Background (BAC –19%), Goal (GOA –1%), Object (OBJ –3%), Method (MET –11%), Experiment (EXP –10%), Model (MOD –9%), Observation (OBS –14%), Result (RES –21%) and Conclusion (CON –9%). Most of these are self-explanatory, however, confusion may arise during annotation especially between the closely related ones, such as GOA and OBJ or OBS and CON. According to [2], an example of a fact (i.e., BAC) is the statement “*AhR agonists suppress B lymphopoieisis*”, while an example of a hypothesis (HYP) is the statement “*the potential of two AhR agonists to alter stromal cell cytokine responses*”.

The multi-dimensional classification corpus [2] consists of 10,000 randomly chosen sentences from random biomedical publications. The provenance of the sentences has not been retained, and hence they are considered stand-alone entities, as opposed to the ART corpus, where sentences appear in the full context of the publication. All sentences have been annotated by three annotators using a scheme tailored to capture the multi-dimensionality of the rhetorical semantics surrounding a sentence. Furthermore, where necessary, sentences have been split into segments (usually clauses) and each segment has been annotated accordingly. The actual scheme comprises:

- the *focus* of the segment, which corresponds to the rhetorical type in the CoreSC scheme – in this case there are three types: Scientific –65%, Methodology –28% and Generic –7%,
- the *polarity*, indicating whether the segment is negative or positive,
- the *certainty*, encoding the degree of certainty associated with the statement,
- the *evidence*, denoting the type of evidence provided in the statement (e.g., no evidence, evidence with no explicit reference or evidence supported by a previous publication), and
- the *direction/trend*, indicating whether an increase or decrease in a specific finding is reported in the segment.

The Scientific type annotates findings and discovery, the Methodology annotates procedures, methods or models, while the Generic annotates general knowledge, e.g., clarifications on the structure of the paper. As an example, the statement “*The binding of both forms of 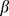-catenin to CBP is completely inhibited by ICG-001 (* *Fig. 3B* *Top, lane 4).*” has the annotation 1SP3E3-, which represents: *Scientific* – *Positive* – *Degree of certainty: 3*– *Experimental evidence is directly given in the text* – *decrease in specific activity/phenomenon*. Complete descriptions of the corpora and the annotation guidelines and inter-annotator agreements can be found in [1], [8], [19], [20] for ART and in [2], [16] for the Wilbur corpus.

**Figure 2:**
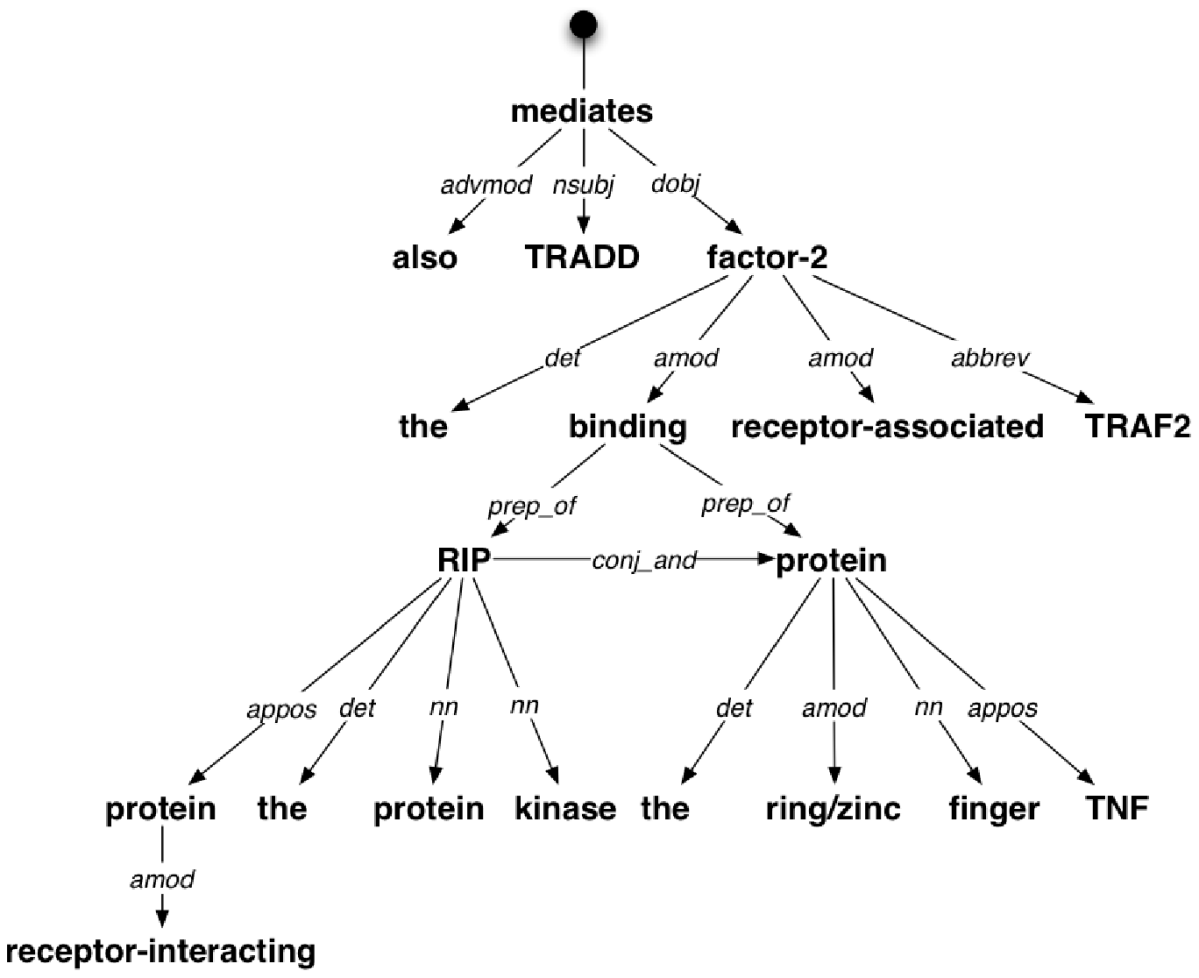
Example of dependency structure. Here we present the graphical representation of the dependency structure of the sentence: “*TRADD also mediates the binding of the protein kinase RIP (receptor-interacting protein) and the ring/zinc finger protein, TNF receptor-associated factor-2 (TRAF2).*”. Nodes in this graph are denoted by the sentence tokens, while edges represent pairwse dependency relations.

**Figure 3:**
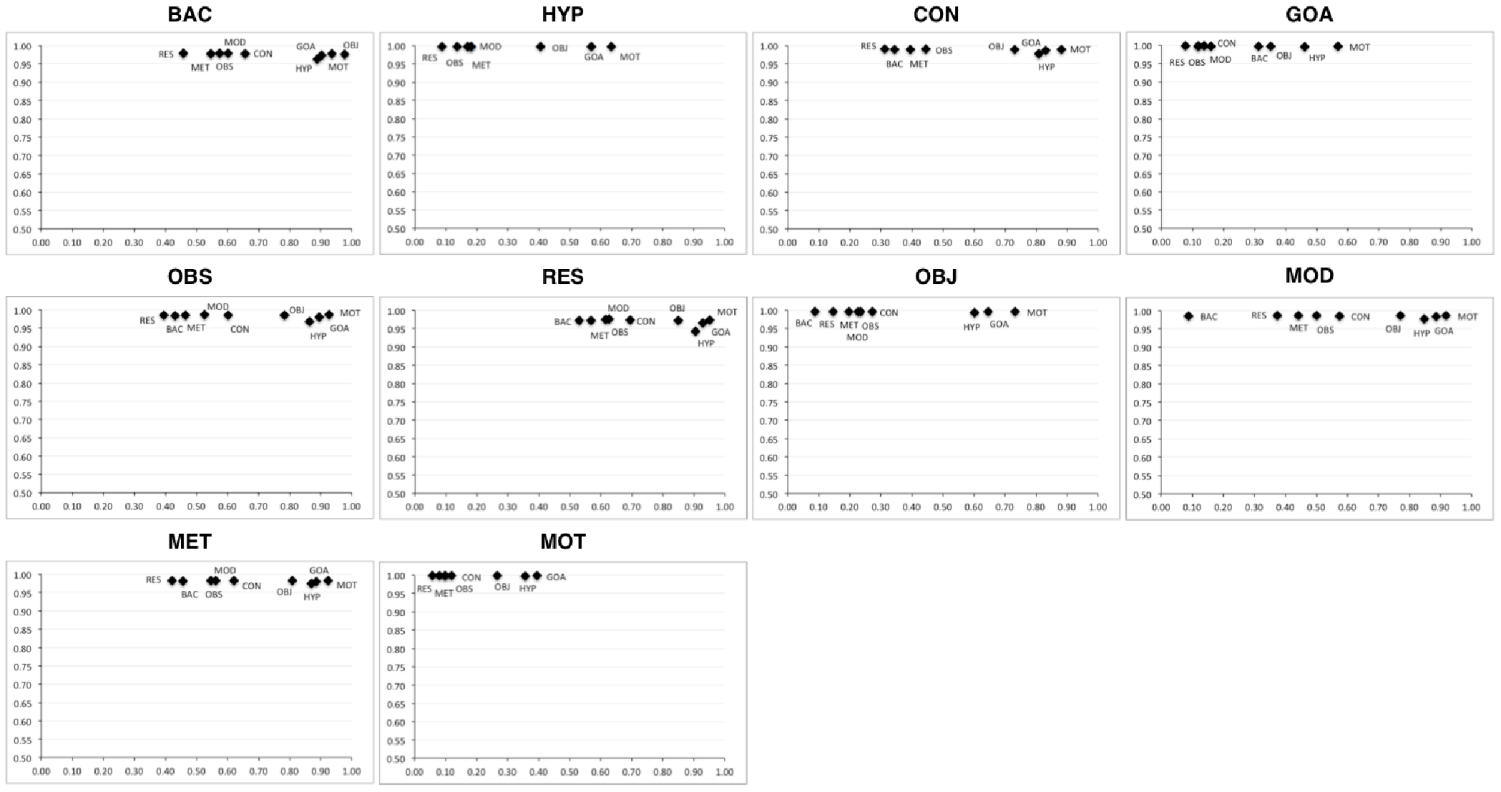
Pairwise sensitivity-specificity graphs for the ART rhetorical types. Each chart represents the sensitivity-specificity graph of a particular rhetorical type, where the X axis denotes sensitivity and the Y axis denotes specificity. The cluster of points closer to the upper right corner of the chars denote rhetorical types against which the type under scrutiny discriminates well (e.g., BAC vs. GOA, HYP, MOT and OBJ).

There are several differences that can be observed between the corpora without taking into account the sizes and distributions of types, however, from our perspective, the most important one is the annotation scheme and granularity. The CoreSC scheme provides a more fine-grained annotation of the rhetorical types, while the multi-dimensional scheme takes into account also statements that do not necessarily have a proper rhetorical relevance, i.e., the Generic type. We discuss in more detail the particular differences between the rhetorical types later in the manuscript. Also, as a remark, due to their increased content in chemical reactions, which results in no (or improper) dependency structures, we have not considered in our experiments the sentences that are part of the EXP ART rhetorical type.

### Dependency Parsing

Dependency parsing, as defined in [21], is the process of automatically analysing the dependency structure of a given sentence. As already introduced, the dependency structure represents a labeled directed graph between the sentence tokens, with the labels – or types – denoting grammatical relationships. These relationships are modelled as triples between pairs of tokens and have the form: *rel*(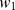, 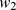), where 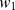 is the governor of the relationship and 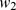 is the dependent. The direction of the relationship points from the governor to the dependent. For example, in the sentence “*TRADD also mediates the binding of the protein kinase RIP (receptor-interacting protein) and the ring/zinc finger protein, TNF receptor-associated factor-2 (TRAF2).*” (part of the Wilbur corpus), the relation “the subject of *mediates* is *TRADD*” is denoted via *nsubj*(mediates, TRADD).

The dependency structure provides a rich insight into the deep semantic relationships between the terms used in the sentence, as shown in an example in Fig. 2. It lays the foundation for building higher-level structures that may then assign a rhetorical meaning to the entire sentence, e.g., sequences or schemes of relationships between segments or sentences via particular theories – see Rhetorical Structure of Text theory [22]. Existing studies, for example the verb study described in [23], or classification approaches, have showed that sentences belonging to the same rhetorical type share a series of linguistic features. This has motivated us to explore whether among these shared characteristics we also find patterns in the dependency structure.

In our experiments, we have used the Stanford parser to generate the dependency structure of the sentences [24]. The Stanford parser defines a hierarchical set of 58 typed dependencies, starting from the most generic grammatical relation – dependent (*dep*) – to more specialised relations, such as, *neg* – negation modifier or *nsubjpass* – passive nominal subject. We refer the reader to [25] for the complete description of these typed dependencies.

Typed dependencies emerging from a sentence intrinsically form a graph structure (Fig. 2 depicts the graph associated with our example sentence). Starting from a virtual relation denoting the root of the sentence the graph is constructed by following the direction of the relation between the governor and dependent. At the same time, the same structure can be represented as a flat list, which follows, to a large extent, the order of the tokens in the sentence. For our example, this list is presented below:

nsubj(mediates-3, TRADD-1) advmod(mediates-3, also-2) root(ROOT-0, mediates-3) det(factor-2-23, the-4) amod(factor-2-23, binding-5) det(RIP-10, the-7) nn(RIP-10, protein-8) nn(RIP-10, kinase-9) prep_of(binding-5, RIP-10) amod(protein-13, receptor-interacting-12) appos(RIP-10, protein-13) det(protein-19, the-16) amod(protein-19, ring/zinc-17) nn(protein-19, finger-18) prep_of(binding-5, protein-19) conj_and(RIP-10, protein-19) appos(protein-19, TNF-21) amod(factor-2-23, receptor-associated-22) dobj(mediates-3, factor-2-23) abbrev(factor-2-23, TRAF2-25).

The two representations are semantically equivalent, however, as we will see in the next section, they may yield completely different results when mining for association rules. In order to understand whether the representation carries a role in the pattern recognition process, we have performed experiments with both the graph structure, as well as with the flat list representation.

### Association Rule Mining

Association rule mining [26] is the process of discovering interesting associations or patterns within large sets of features, in principle, by considering the features that occur frequently together. For example, in a diagnosis context, the association rule {Short stature, Macrocephaly} 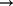 {Achondroplasia} may imply that if both {Short stature} and {Macrocephaly} are patient phenotypes, then the patient is likely to have Achondroplasia – subject to the strength of the rule in the given dataset.

Association rules externalise knowledge in the form of probabilistic “if-then” statements. The head of the rule is called *antecedent*, while the body of the rule is called *consequent*. The antecedent and consequent of an association rule are always disjoint. The uncertainty, or strength, of an association rule is usually expressed via two quantifiers: (i) *support*, and (ii) *confidence*. The *support* of a rule is defined as the total number of transaction (in our case sentences) that include all items (in our case dependency relations) in the antecedent and consequent (Eq. 1). *Confidence*, on the other hand, represents the ratio between the total number of transactions that include the items of both the antecedent and the consequent and the number of transactions that include only the items in the antecedent (Eq. 2) [27].
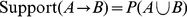
(1)

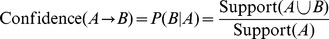
(2)


Over the course of the last 20 years, there have been a wide range of algorithms proposed for mining association rules. Within our experiments, we have employed one of the most popular algorithms, i.e., Apriori [26].

### Methodology

To reiterate, our goal is to use the dependency structure of the sentence in the process of discovering patterns of typed dependencies corresponding to the rhetorical types defined by the corpus under scrutiny. At the same time, using these patterns, we aim to explore their discriminative power, i.e., to what extent are they able to classify sentences according to the defined rhetorical types. In order to achieve this, we have employed the following process:

- **Mining association rules from dependency structures:** we parsed all sentences in both corpora, grouped by their rhetorical type, and used the typed dependencies to mine group-based association rules. For example, by taking into account all RES sentences from the ART corpus, we mined the association rules that characterise this rhetorical type.
- **Defining rule interestingness measures:** we used a series of composite metrics to filter the resulting rules. As we discuss below, the process of mining association rules usually leads to an immense rule set. The challenge here is to filter those rules that describe better the underlying rhetorical type.
- **Defining the classification mechanism:** we defined a way to compute rule covers, i.e., a measure that shows to what extent a rule matches a given dependency structure, in addition to a set of aggregation mechanisms to compute the overall probability that an unseen sentence will be tagged with a rhetorical type, given the set of rules corresponding to that type.

#### Mining association rules from dependency structures

In a previous section we have shown how we can represent a dependency structure: using the intrinsic graph representation (depicted in Fig. 2) or the flat list representation based on the token order in the sentence. Any of these representations can be serialised in a format usable as input in the association rule mining process. More concretely, there are two serialisation options: (i) as a set, or (ii) as a sequence.

The set-based serialisation treats the dependency structure as a bag of unique typed dependencies, i.e., duplicated elements are removed and the order is discarded. In this case, both representations lead to the same serialisation. Additionally, we have introduced two further simplifications: we removed the root relation from the serialisation (since it introduces no variance in this setting) and we merged the different instances of the prep and conj relations (e.g., prep_of or prep_in and conj_and or conj_or) under their generic type. For example, returning to the sentence introduced earlier in the article (“*TRADD also mediates the binding of the protein kinase RIP (receptor-interacting protein) and the ring/zinc finger protein, TNF receptor-associated factor-2 (TRAF2).*”), the resulting set serialisation will be: {nsubj, advmod, det, amod, nn, prep, amod, appos, conj, dobj, abbrev}. It can be observed that relations such as nn or det are listed only once and the order is irrelevant. The intuition here is that each rhetorical type will manifest a slightly different set of typed dependencies, without taking into account their order.

In contrast to the set-based serialisation, the sequence-based serialisation maintains both the entire set of typed dependencies, as well as their order. Consequently, the two possible representations of the dependency structure will lead to different serialisations. Furthermore, we have the option of grouping several elements and representing the sequence using these groups, rather than the individual elements. As a note, in this case the order is relevant at group level, i.e., between the groups, and not at individual level, i.e., within the groups. As mentioned previously, sequences of dependency relations may form higher-level structures that capture the rhetorical meaning of a sentence. However, there are no guidelines that may lead us to such patterns. Hence, the only possible option is to explore the effect of grouping typed dependencies via different schemes. As a result, we have experimented with four grouping strategies (examples are provided using the same sentence – also, similar to the set-based serialisation, the different instances of the prep and conj relations are merged under the generic type):

- *bag-of-1* sequence: starting from the flat representation, we treated each typed dependency as a group, i.e., (nsubj), (advmod), (root), (det), (amod), (det), (nn), (nn), (prep), (amod), (appos), (det), (amod), (nn), (prep), (conj), (appos), (amod), (dobj), (abbrev)
- *bag-of-2* sequence: as above, but grouping is performed between every two consecutive typed dependencies, i.e., (nsubj, advmod), (root, det), (amod, det), (nn, nn), (prep, amod), (appos, det), (amod, nn), (prep, conj), (appos, amod), (dobj, abbrev)
- *bag-of-3* sequence: as *bag-of-2*, however using three consecutive typed dependencies, i.e., (nsubj, advmod, root), (det, amod, det), (nn, nn, prep), (amod, appos, det), (amod, nn, prep), (conj, appos, amod), (dobj, abbrev)
- *BF-bag* sequence: using the graph representation, we performed a custom breadth-first traversal by touching each node once, to avoid cycles, and by forming bags of siblings (instead of bags containing all elements) at each level, i.e., (nsubj, advmod, dobj), (det, amod, amod, abbrev), (prep, prep), (appos, det, nn, nn), (det, amod, nn, appos), (amod). The root relation is again removed because it shows no variance – all graph representations start with the virtual ROOT node.

By using sequences, we are trying to understand whether the rhetorical types are governed by structural patterns, or more concretely, whether the way in which sentences are constructed plays a role in defining the rhetorical meaning of the sentence.

As part of a general nine-fold cross-validation process, we ran the association rule mining algorithm on both corpora and performed an initial filtering on the resulting rules, by removing all rules with a support less than 5%. For clarification, an association rule mined via this process will have the form: {det, amod, nsubj} 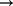 {dobj, abbrev}, irrespective of the format of the input data (i.e., set-based or sequence). Tables 1 and 2 list the average number of rules extracted from each corpus, according to the rhetorical type and with a support greater than 5%.

**Table 1:** Rule statistics on the Wilbur corpus.

Rhetorical type	No. sentences	Set-based	Bag-of-1	Bag-of-2	Bag-of-3	BF-Bag
Generic	639	86,326	303,426	240,268	181,924	5,548
Methology	2,694	458,855	749,899	590,100	472,021	17,211
Scientific	6,190	470,845	967,994	724,746	542,111	16,887

Average number of rules generated by the association rule mining process for each of the five types of serialisation of the dependency structure, on the Wilbur corpus.

**Table 2:** Rule statistics on the ART corpus.

Rhetorical type	No. sentences	Set-based	Bag-of-1	Bag-of-2	Bag-of-3	BF-Bag
BAC	5,784	2,055,686	157,404	125,085	99,203	58,069
CON	3,626	446,616	33,771	25,427	19,050	10,231
GOA	580	447,576	41,219	31,566	24,183	13,289
HYP	785	764,476	37,117	27,467	20,243	11,433
MET	3,891	586,364	34,374	26,245	19,762	11,714
MOD	3,856	498,224	28,106	21,847	16,880	10,868
MOT	484	542,630	40,035	29,692	23,550	10,065
OBJ	1,172	147,610	13,498	10,088	7,908	4,553
OBS	6,110	48,538	4,223	3,108	2,276	1,826
RES	8,891	213,616	18,470	13,898	10,579	6,365

Average number of rules generated by the association rule mining process for each of the five types of serialisation of the dependency structure, on the ART corpus.

We can observe that there is no particular relation between the number of sentences and the number of generated rules. For example, MOD in ART has 3,856 sentences and a rule set of almost 500K rules, while MOT has only 484 sentences, hence an order of magnitude less, but the rule set has similar size – over 500K rules. The only observable, and natural, pattern is the negative association between the number of rules and the restrictions imposed by the different serialisation: the more restrictions a serialisation adds, the fewer rules are produced. This is, however, not completely respected by the Wilbur corpus. The set-based serialisation, which imposes no order restrictions, has produced fewer rules than the initial sequence-based serialisation, and sometimes fewer than all except the *BF-Bag* – see the *Methodology* type. This could be related to the size of the set in the set-based serialisation. While the length of the sentences in the two corpora are similar, i.e., there is no clear indication that the Wilbur corpus features shorter sentences, the size of the resulting set-based serialisation is smaller on average by 20%. Since this is dictated by the number of unique typed dependencies, it seems that the sentences in the Wilbur corpus contain more duplicates than the ones in the ART corpus. A different reason may be related to the overall number of typed dependencies present in a rhetorical type over the imposed 5% support threshold, e.g., in the *Generic* type only 23 of the 58 typed dependencies have a support over 5%. However, this is also influenced by the number of sentences corresponding to that rhetorical type, and hence cannot be directly accounted as the main factor.

Taking into account that more rules reflects a more diluted language structure, we can draw the following conclusions:

- within the ART corpus, at the first glance, the BAC rhetorical type appears to be the least consistent. However, at a more careful look, judging by the association number of sentences – number of rules, the HYP, GOA, and MOT are particularly diluted. At the opposite side, OBS and RES are governed by a more restrained set of rules.
- between the two corpora, the dependency structure of the sentences in the Wilbur corpus seems to be much more diverse (see the number of rules generated from the sequence-based serialisations), which may be associated with the fact that the sentences have been randomly selected from random publications from multiple domains.

Below we list some examples of “interesting” rules that contain “rare” typed dependencies with high *confidence* values:

- HYP (set-based): {*conj*, *amod*, *advcl*, *det*, *advmod*, *prep*} 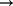 {*nsubj*} (93.33%)
- GOA (tree): {*dep*, *aux*, *auxpass*} 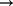 {*amod*} (81.39%)
- Scientific (tree): {*advcl*} 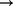 {*mark*} (81.59%)
- Methodology (set-based): {*dobj*, *nn*, *num*, *number*} 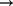 {*amod*} (91.42%)

#### Rule interestingness measures

As seen in the previous section, the size of the resulting rule set, even for rhetorical types under-represented in the corpus, is considerable. This makes the discovery of the relevant patterns particularly challenging. It is, however, a typical challenge associated with the rule mining process, because the *support* and *confidence* are, on their own, not enough to define the “interestingness” of a rule [27]. Rules with high support are, in principle, not interesting because they represent common knowledge. On the other hand, rules with high confidence are desirable, however, filtering only on confidence might not necessarily reduce the initial rule space significantly.

Finding good rule interestingness measures, useful as filtering mechanism, has been a hot research topic in the Data Mining field [27]. Among the proposed measures, the most widely used have been 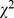, which relies on the 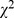 statistics of computing the difference between the observed and expected values or *lift*, which tests the independence of occurrence between the antecedent and the consequent of the rule, i.e., 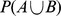 vs. 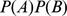. We refer the reader to [27] for a comprehensive description and discussion of rule interestingness measures. On the other hand, it has been demonstrated that most of the proposed measures suffer from sensitivity to the size of the data not covered by the union of the antecedent and consequent, i.e., the entire rule, which in many typical scenarios is huge and unstable.

One measure that has been shown to deal with this issue is the Kulczynski measure, usually abbreviated as *Kulc* – defined in Eq. 3. This represents the average between the confidence of the antecedent and the confidence of the consequent, and is hence independent of the support, which is the root of the above-mentioned issue. Furthermore, it has been suggested that, in order to avoid a possibly skewed relation between the antecedent and consequent, i.e., 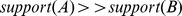, *Kulc* should be used in conjunction with an *imbalance ratio*, or *IR* – defined in Eq. 4, which assesses the imbalance of the underlying item sets of the antecedent and consequent [27].
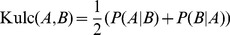
(3)

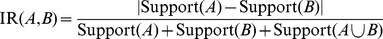
(4)


Taking as input the rules produced in the previous step, we experimented with using *support*, *confidence*, *IR* and *Kulc* as rule interestingness measures. In total, we have designed four rule rankings based on these measures and applied different thresholds with the goal of finding the combination that leads to the most interesting patterns. The rankings have been computed using: (i) ordinary *support*, (ii) ordinary *confidence*, (iii) a filter based on *IR* with a range of 40%–60%, i.e., the almost perfectly balanced rules, followed by ranking based on *Kulc*, and (iv) the same filter as above, but followed by plain *confidence*. The thresholds used within the experiments have been: 1%, 5%, 10% and 15%. Table 3 lists an example of number of rules resulting from applying the *support* and *IR – Kulc* measures, together with a threshold of 5%, on the ART corpus. Conforming to our expectations the *IR – Kulc* yielded more rules than *support*. This is because a large proportion of rules in the *IR – Kulc* set have the same *Kulc* value, as opposed to the rules ranked by *support*, which rarely share their value. Complete statistics of the rule sets are provided in Table sets S1 and S2.

**Table 3:** Example of rule filtering using two ranking methods.

Rhetorical type	Support					IR – Kulc				
	Set	Bag-of-1	Bag-of-2	Bag-of-3	BF-Bag	Set	Bag-of-1	Bag-of-2	Bag-of-3	BF-Bag
BAC	498	103	97	90	81	20,560	1,028	809	646	414
CON	350	68	65	62	49	4,406	238	181	141	78
GOA	62	17	17	18	15	4,000	294	226	175	96
HYP	72	26	20	18	16	7,106	256	193	145	86
MET	328	71	60	55	51	5,874	242	191	646	91
MOD	264	58	57	50	46	4,794	211	166	132	94
MOT	54	14	14	12	11	5,172	271	205	167	78
OBJ	74	27	27	24	19	1,516	99	78	63	36
OBS	220	53	49	43	37	532	39	30	24	17
RES	484	108	100	91	75	2,344	140	110	85	55

Top 5% rule counts according to the dependency structure serialisation for *support* and *IR – Kulc* measures in the ART corpus.

One aspect that is worth reiterating here is that association rule mining targets those items that are frequently occurring in the data. In the case of natural language, irrespective of the underlying corpus, we would expect typed dependencies such as *det*, i.e., determiner – “*the* protein”, or *prep*, i.e., preposition – “binding *of* RIP”, to have the highest individual supports. The analysis of our two target corpora demonstrates this aspect. For example, in BAC *prep* has a support of 93.67%, while *det* a support of 91.21%, i.e., the top two in the individual support ranking, and a similar ranking is present in the other rhetorical types of both the ART and Wilbur corpora. In order to study the effect of these typed dependencies on the quality of the resulting rules, we have repeated the filtering steps above and removed the rules that contain these relations. This has led to an average reduction in the number of rules of one order of magnitude. Again, complete statistics are provided in Table sets S1 and S2.

Overall, our experiments have been performed on 40 rule sets for each rhetorical type, for each ranking threshold in the context of a single corpus: 5 serialisation types × 4 interestingness measures × 2– with and without determiners and prepositions. In total, we have analysed 160 rule sets per rhetorical type in each corpus.

#### Rule aggregation mechanisms for classification

The rules extracted via the mechanisms described so far enable intra- and inter-corpus analysis. More concretely, they allows us to study differences in patterns between the rhetorical types within a corpus, as well as between the two corpora. In order to explore their discriminative power, an additional experiment is required, i.e., to use the rules mined from the dependency structure to attain the same goal as the approaches described in [1] and [2]: recognition of conceptualisation zones, or sentence-based classification according to a given schema.

Two additional elements are required to enable classification: (i) a *rule cover*, and (ii) a *score aggregation mechanism*. Given the dependency structure of a sentence and an association rule, the *rule cover* quantifies the rule's ability to match the dependency structure, or more concretely, how many items in the rule match the dependency structure. A widely adopted measure to compute the rule cover is the Jaccard index (or Jaccard similarity coefficient). In general, the Jaccard index is defined as the ratio between the intersection and the union of two sets. In practice, this takes various forms subject to the requirements of the computed similarity. In our case, rules have an antecedent and a consequent (disjoint) and the goal is to quantify to what extent the rule is matched. We, hence, adapted the Jaccard index to define the rule cover as the average between the coverage of the antecedent and the coverage of the consequent, both with respect to the given sentence (see Eq. 5).
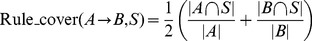
(5)


One particular remark related to the rule cover is that, applied under the general set assumptions, it does not take order into account. This works, as envisioned, for the association rules mined from the set-based representation of the dependency structure, however, it requires a special implementation for the association rules resulted from the other representation. For example, given the example sentence used so far in this article with the serialisation (nsubj, advmod, root, det, amod, det, nn, nn, prep, amod, appos, det, amod, nn, prep, conj, appos, amod, dobj, abbrev) and the rule {*nsubj*, *amod*, *advmod*, *nn*} 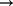 {*dobj*, *appos*}, the cover is computed subject to the provenance of the rule. Hence, if the rule has resulted from a set-based representation, Rule_cover(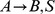) = 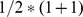 = 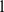 (all rule items are matched). However, if the rule has resulted from any of the sequence-based representations, then Rule_cover(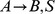) = 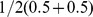 = 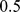 (in the antecedent the third item breaks the order, and hence only the first two provide a match; similarly also for the consequent). This difference in computation forces the rule cover to reflect the underlying nature of the rule, and more importantly, the need to maintain, or not, the order.
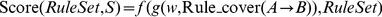
(6)


The second element required to enable classification is a way to aggregate the individual scores (or covers) of a set of rules corresponding to a rhetorical type. More concretely, given the dependency structure of a sentence (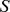) and the rule set of a rhetorical type, e.g., BAC in ART (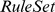), we need to define the probability of that sentence belonging to the rhetorical type. In general, this score can be defined as in Eq. 6, where (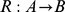) is a rule, 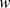 is a weight, 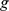 is a function that aggregates the weight 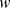 and the cover of the rule 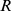, and 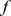 is a function that aggregates the individual 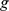 values of the rules 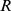 in 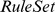. We, hence, have three parameters that can be exploited. Within our experiments, we have used the following values:


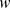
 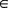

  1 (i.e., no weight)
*support*
*confidence*
*Kulc*

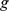
 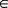

  multiplication – 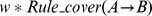

  arithmetic mean – 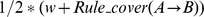

  harmonic mean – 
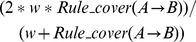

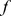
 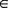

  mean
  max
  mean of elements in the range [−SD, +SD] (where SD is the standard deviation of the scores across all rules)

The combination of the three variables (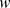, 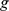 and 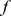), each of which may take four, three and three values, respectively (see listing above) results in 36 ways of computing the final aggregation score. In our experiments we have used all these 36 possibilities, however, below we present and discuss only the combinations that led to optimal values.

## Results

### Corpus Analysis

As mentioned previously, unlike typical ML features, rules enable a comparative study of the rhetorical types, within and across corpora. More specifically, using the four ranking methods we have proposed, we were able to analyse the overlap in rules in the context of a given serialisation between rhetorical types, and hence in the style of the dependency structures, between serialisations in the context of a given rhetorical type, as well as between the rhetorical types of our two corpora. As a note, the comparison between two rule sets is uni-directional because they rarely have the same size and content. Below we summarise the key findings, and refer the reader to Table sets S3, S4, S5, S6, S7 for the raw data.

#### Comparison between rhetorical types

The different ranking methods provide different perspectives on the degree of overlap between the rule sets. However, this overlap is highly dependent on the size of the rule set used as reference, which makes the comparative analysis difficult. For example, Background (BAC) in ART has 100K+ rules in the set-based serialisation filtered by confidence in top 5% vs. 46K of Hypothesis (HYP). Similarly, the rule sets of the Generic type in the Wilbur corpus are usually a third in size when compared to the other types.

Nevertheless, we were able to observe the following aspects – independently of the serialisation type and ranking mechanism:

- The overall percentage of overlap between the types and the bi-directional overlap ratio between any two particular types is uniform across the number of rules considered for analysis. For example, Background (BAC) and Hypothesis (HYP) in ART share a proportionally constant number of rules, indifferently on the initial rule set size, i.e., top 1%, 5%, 10% or 15%. This leads to the conclusion, later supported also by the classification experiments, that a small number of rules – in principle, up to top 5% – is enough to explain the patterns present in the dependency structures.
- Eliminating the highly occurring typed dependencies, i.e., *det* and *prep*,in the sequence-based serialisations leads to an up to 50% increase in overlap. Hence, indifferently on the underlying rhetorical type, these typed dependencies carry an important role in diversifying the dependency structure of a sentence, and in practice, their absence decreases the discriminative power of the resulting rule set.
- Most rhetorical types are well-defined. This observation is supported by any of the ranking mechanisms except the support-based, which provides poor filtering capabilities. The only exceptions are Observation (OBS) and Object (OBJ) in ART, due to their rule set size. In other words, between any two types in the same corpus, the rules emerging from their corresponding dependency structures have an overlap of maximum 50% in the case of the ART corpus and maximum 30% in the Wilbur corpus. This analysis shows, from a quantitative perspective, that there is a relationship between the structure of the sentences and their rhetorical definition (seen much better in the Wilbur corpus due to the coarser granularity of the rhetorical types) and that the underlying dependency structure is different even for rhetorical types that are usually hard to distinguish – for example, Goal (GOA) and Object (OBJ) in ART.

#### Comparison between serialisations

In the Methodology section we have shown that the dependency structure can be serialised in several ways, and in the context of our experiments, we have focused on five such serialisations: set, and four types of bags – *Bag-of-1*, *Bag-of-2*, *Bag-of-3* and *BF-Bag*. The rationale behind studying multiple types of serialisation was to explore whether the resulting rule sets are different. Table sets S5 and S6 lists the complete comparative analysis between the different serialisations. Below, we focus on two findings that emerge from this comparison – supported by all ranking mechanisms:

- The degree of overlap between rule sets decreases with the decrease in restrictions imposed by the serialisation mechanism. More concretely, *Bag-of-3* has a higher overlap to *Bag-of-2* than to *Bag-of-1* across all rhetorical types. This is contrary to our expectation, since we assumed more restrictions to lead to rules already covered by the less restrictive serialisations.
- As presented in Table 4, among the ranking mechanisms, *IR-Kulc* delivers the best diversity, in the ART corpus, with an average standard deviation of 0.22 between the different serialisations across all rhetorical types. Similarly, *confidence* enables a greater diversity, in the Wilbur corpus, with an average standard deviation of 0.37. Our assumption is that the diversity provided by these two ranking mechanisms will increase the discriminative power of the corresponding rule sets in the corpus-driven classification.

**Table 4:** Cross-type comparison within each corpus based on the different ranking mechanisms.

Ranking mechanism	ART	Wilbur
Confidence	0.17	**0.37**
Support	0.18	0.22
IR-Confidence	0.19	0.25
IR-Kulc	**0.22**	0.29
IR-Mixed	0.19	0.31

Average standard deviations of the overlaps across different serialisations in the top 5% of the rules in both corpora. Bolded values denote the ranking mechanisms that deliver the best diversity within each corpus – i.e., the larger the average standard deviation, the better the cross-type diversity.

#### Comparison between corpora

As opposed to standard ML features, rules also allow us to perform a comparative analysis of the underlying structures of the rhetorical types across multiple corpora, which may provide the foundation for creating appropriate mappings between them. A direct mapping is infeasible, due to the difference in granularity. Nevertheless, according to the definitions provided in the annotation guidelines of the two corpora, we would expect a fairly high similarity between the Wilbur Methodology and ART Method (MET) and Model (MOD) types, and between the Wilbur Scientific type and ART Result (RES), Observation (OBS) and Conclusion (CON) types. Subject to the style of writing, one may see feasible mappings also between the ART Goal (GOA) and Hypothesis (HYP) types Wilbur's Scientific type.

These mapping patterns are, however, only partly revealed by the data via the *confidence* and *support* based rankings and independently on the inclusion or exclusion of the *det* and *prep* typed dependencies – see Table 5. There are three observations worth noting here:

**Table 5:** Cross-corpus comparison overview.

	Overlap from ART to Wilbur types			Overlap from Wilbur to ART types		
	Generic	Methodology	Scientific	Generic	Methodology	Scientific
BAC	0.05	0.11	0.20	0.72	**0.41**	0.69
CON	0.17	0.17	**0.70**	0.66	0.18	**0.70**
GOA	0.18	0.27	0.34	**0.83**	0.34	0.37
HYP	0.12	0.13	0.45	0.75	0.22	**0.70**
MET	0.16	**0.34**	0.43	0.70	**0.43**	0.45
MOD	0.13	0.28	0.47	0.56	**0.33**	0.48
MOT	0.18	0.20	0.47	0.74	0.21	0.47
OBJ	**0.41**	**0.52**	**0.72**	0.57	0.20	0.24
OBS	**0.59**	**0.50**	**0.87**	0.20	0.05	0.07
RES	0.29	0.30	**0.83**	0.44	0.13	0.30

Cross-corpus overlap in rule sets using the *confidence* ranking mechanism and the *set-based* serialisation. Bolded values denote the highest rule sets overlap between the corresponding types and are indicative of possible cross-corpus type mappings.

- The set-based serialisation shows a bi-directional overlap of 70% between Scientific and Conclusion (CON), 35% between Scientific and Goal (GOA) and 45% between Scientific and Hypothesis (HYP). Similarly, there is an overlap of around 40% between Methodology and Method (MET) and around 30% between Methodology and Model (MOD).
- The sequence-based serialisations, on the other hand, no longer confirm these mappings, due to the difference in the size of the rule set. As shown in Tables 1 and 2 the number of rules generated from the Wilbur corpus using sequence-based serialisations is, on average, larger by almost one order of magnitude than the one generated from the ART corpus.
- The Wilbur Generic type has a considerable overlap, on average more than 30%, with most of the ART types, while the Scientific type seems to be very similar to both Method (MET) and Model (MOD), with a bidirectional overlap of around 46%. As we will see in the cross-corpus classification, these mappings will affect the overall classification results, for example, by making it impossible for any of the ART types to make a difference in classifying the Wilbur Generic type.

### Corpus-driven Classification

In the previous section, we have shown the degree of similarity between the rhetorical types within a corpus and between the rule sets generated via diverse serialisations. This, however, does not reveal to what extent are the corresponding rule sets discriminative. Consequently, we have performed corpus-driven classification using the rule cover and aggregation mechanisms described in the Methodology section. The classification process had the following steps:

- we took each individual sentence in each corpus, i.e., sentence-based classification,
- we scored the sentence according to each rule set associated to each rhetorical type in the underlying corpus, i.e., we have computed the probability that the sentence is part of a particular rhetorical type – e.g., Background (BAC) in ART – given its corresponding rule set, and finally,
- we ranked the scores, and hence, created a ranking of the most probable rhetorical types.

In order to make our results comparable to those of Liakata and Wilbur, we performed a nine-fold cross validation and report here the averages of the standard macro precision, recall and F1.

Table 6 lists the best results achieved for the ART corpus at rank K = 1, 2 and 3, while Table 7 lists the best results achieved for the Wilbur corpus at rank K = 1. Please note that the choice of levels for K was driven by the number of rhetorical types featured by the corpora. Hence, since ART comprises 10 rhetorical types, an analysis of up to the top three best ranked candidates is relevant. However, in the case of the Wilbur corpus, the annotation scheme uses only three types, and thus the only relevant option is to consider the rhetorical type with the highest probability, i.e., top-1. Both tables, 6 and 7, show the results computed based on the top 1% and 5% of the rules, including and excluding the frequent typed dependencies *det* and *prep*. In the case of the ART corpus the parameters that led to the best results were:

**Table 6:** Corpus-driven macro classification results – the ART corpus.

		Top 1% of the rules						Top 5% of the rules					
		Including *det* and *prep*			Excluding *det* and *prep*			Including *det* and *prep*			Excluding *det* and *prep*		
		P	R	F1	P	R	F1	P	R	F1	P	R	F1
K = 1	BAC	0.18	0.49	**0.27**	0.18	0.57	**0.27**	0.18	0.49	**0.27**	0.18	0.52	**0.27**
	CON	0.10	0.41	0.16	0.11	0.85	**0.20**	0.10	0.41	0.16	0.11	0.86	0.20
	GOA	0.02	0.59	0.04	0.02	0.50	0.04	0.02	0.59	0.04	0.02	0.57	0.04
	HYP	0.02	0.87	0.05	0.02	0.45	0.05	0.02	0.87	0.05	0.02	0.44	0.05
	MET	0.16	0.61	**0.25**	0.15	0.61	**0.24**	0.16	0.61	**0.25**	0.15	0.60	**0.25**
	MOD	0.13	0.46	**0.20**	0.13	0.46	**0.20**	0.13	0.46	0.20	0.13	0.47	0.20
	MOT	0.01	0.40	0.03	0.01	0.42	0.03	0.01	0.40	0.03	0.01	0.39	0.03
	OBJ	0.04	0.48	0.07	0.03	0.43	0.06	0.04	0.48	0.07	0.03	0.42	0.06
	OBS	0.14	0.33	0.19	0.14	0.34	0.20	0.14	0.32	0.19	0.14	0.33	0.20
	RES	0.21	0.33	0.26	0.21	0.34	0.26	0.21	0.33	0.26	0.21	0.34	0.26
K = 2	BAC	0.23	0.21	0.22	0.17	0.71	**0.28**	0.34	0.09	0.14	0.21	0.15	0.18
	CON	0.15	0.45	**0.23**	0.11	0.93	**0.20**	0.19	0.28	**0.23**	0.18	0.29	**0.22**
	GOA	0.06	0.30	**0.10**	0.02	0.51	0.04	0.05	0.33	**0.09**	0.04	0.45	0.07
	HYP	0.06	0.18	**0.09**	0.02	0.48	0.05	0.09	0.12	**0.10**	0.04	0.15	0.06
	MET	0.14	0.02	0.03	0.15	0.63	0.24	0.20	0.14	0.17	0.11	0.07	0.09
	MOD	0.18	0.13	0.15	0.12	0.53	0.20	0.18	0.11	0.14	0.13	0.18	0.15
	MOT	0.02	0.49	**0.05**	0.01	0.51	0.02	0.03	0.36	0.05	0.02	0.06	0.03
	OBJ	0.05	0.41	**0.09**	0.04	0.48	0.07	0.05	0.41	0.09	0.05	0.42	0.09
	OBS	0.20	0.61	**0.30**	0.15	0.36	0.21	0.22	0.75	0.34	0.20	0.68	0.31
	RES	0.34	0.32	0.33	0.24	0.58	0.34	0.30	0.57	0.39	0.30	0.46	0.37
K = 3	BAC	0.21	0.39	**0.28**	0.16	0.68	0.25	0.29	0.13	0.18	0.22	0.26	0.24
	CON	0.14	0.59	**0.23**	0.11	0.37	0.17	0.15	0.45	**0.23**	0.15	0.62	**0.24**
	GOA	0.05	0.37	0.09	0.03	0.45	0.05	0.05	0.37	**0.08**	0.04	0.48	0.07
	HYP	0.05	0.34	**0.09**	0.01	0.05	0.02	0.06	0.17	**0.09**	0.06	0.25	**0.10**
	MET	0.16	0.04	0.07	0.12	0.19	0.15	0.20	0.39	**0.26**	0.19	0.42	**0.27**
	MOD	0.15	0.32	**0.21**	0.11	0.75	**0.20**	0.16	0.23	0.19	0.15	0.42	0.22
	MOT	0.02	0.59	0.04	0.02	0.27	0.03	0.02	0.58	0.04	0.02	0.68	0.04
	OBJ	0.05	0.58	0.08	0.02	0.04	0.03	0.04	0.50	0.08	0.04	0.45	0.08
	OBS	0.20	0.67	**0.31**	0.32	0.12	0.17	0.20	0.84	0.33	0.26	0.43	0.33
	RES	0.32	0.48	**0.38**	0.26	0.79	**0.39**	0.29	0.69	0.41	0.30	0.56	0.39

Classification results – macro-precison/recall/F1– for the ART corpus using top 1% and 5% of the rules and including and excluding the *det* and *prep* typed dependencies. Bolded values denote the highest F1 scores achieved by the corresponding type within a particular category of results. For example, in the top 1% of rules that include the *det* and *prep* typed dependencies, the ART Method type (MET) has achieved the highest F1 of 0.25, for K = 1.

**Table 7:** Corpus-driven macro classification results – the Wilbur corpus.

		Top 1% of the rules						Top 5% of the rules					
		Including *det* and *prep*			Excluding *det* and *prep*			Including *det* and *prep*			Excluding *det* and *prep*		
		P	R	F1	P	R	F1	P	R	F1	P	R	F1
K = 1	Generic	0.14	0.15	0.14	0.10	0.35	0.15	0.11	0.30	**0.16**	0.12	0.30	**0.17**
	Methodology	0.61	0.68	0.64	0.57	0.75	**0.65**	0.56	0.76	**0.65**	0.57	0.75	0.64
	Scientific	0.80	0.85	**0.83**	0.79	0.87	**0.83**	0.80	0.85	0.82	0.82	0.79	0.80

Classification results – macro-precison/recall/F1– for the Wilbur corpus using top 1% and 5% of the rules and including and excluding the *det* and *prep* typed dependencies. Similar to Table 6, the highest F1 scores achieved by a type within a particular category of results are represented by the bolded values.

- for K = 1, the IR-Mixed ranking of the set-based serialisation with *w = confidence*, *g = arithmetic mean* and *f = mean*,
- for K = 2, the IR-Mixed ranking of the set-based serialisation with *w = confidence*, *g = harmonic mean* and *f = mean of standard deviations*,
- for K = 3, the confidence ranking of the set-based serialisation with *w = confidence*, *g = harmonic mean* and *f = mean of standard deviations*,

Similarly, for the Wilbur corpus the parameters leading to the best results were:

- for top 1%, the confidence ranking of the *Bag-of-3* serialisation with *w = support*, *g = multiplication* and *f = mean of standard deviations*,
- for top 5%, the IR-Kulc ranking of the *BF-Bag* serialisation with *w = support*, *g = arithmetic mean* and *f = mean of standard deviations*,

In the case of the ART corpus, the results present two findings:

- the classification efficiency improves with the increase of K only for some of the rhetorical types, e.g., Result (RES) – from 0.26 to 0.38 F1 or Observation (OBS) – from 0.19 to 0.31 F1. This leads to the conclusion that the patterns defined by the other types are rich enough to improve the overall discriminative power of the rule set.
- some of the rhetorical types “dominate” the classification, hence the assumption that they are supported by clearly established patterns – e.g., Background (BAC): 0.27 F1, Method (MET): 0.25 F1 or Result (RES): 0.26 F1 in the top 1% of the rules.

The Wilbur corpus, on the other hand, displays better classification results, in principle, due to the coarser granularity of the rhetorical types. As shown in an earlier section, these are much better defined, i.e., the overlap in rules between the types is considerably lower. Here, both the Scientific (0.83 F1) as well as the Methodology (0.63 F1) types achieve good classification results using both the top 1% or top 5% of the rules. The low score achieved by Generic, which performed the lowest also in the original experiments of Wilbur, was expected, since by definition this type collates all other sentences that do not fit into the two main target types.

### Cross-corpus Classification

The corpus-driven (or direct) classification presented earlier represents the classic experiment performed by all other existing approaches. In this section, we aim to study to what extent we can use the rule sets associated with the ART rhetorical types to classify Wilbur types. We have seen in the cross-corpus comparative analysis that there is some overlap between the types that have been defined in a fairly similar manner – if we abstract from the large granularity difference. Hence, the challenge is to test whether the rule sets supporting this comparative analysis are also discriminative enough.

In order to perform the cross-corpus classification, we have used the following process: (i) for each sentence in the Wilbur corpus, using each rule set associated with the ART rhetorical types, (ii) we computed the score of the sentence being part of the corresponding ART type, (iii) we re-arranged the sentences according to the Wilbur types, and finally we introduced a variable threshold over the ART scores, which is then used to delimit the ART types that assign a higher score to the underlying Wilbur type. The ART types assigning a score over the threshold are considered in the computation of standard macro precision. The intuition here is that if an ART type is able to discriminate between the different Wilbur classes, then it will assign higher scores for that type, and consequently, by increasing the threshold, the precision for that class will be higher while at the same time lower for the rest of the types.

Table 8 lists three types of results achieved for the best classifier using the top 1% of the ART rules in any sequence-based serialisation and diverse score aggregation mechanisms. As it can be observed, except for the second result set, where there is a clear separation between Method (MET) and the rest, in the classification of the Methodology type, there are only slight indications that the pairs of types we have previously discussed are actually forming. In both the first and third result sets all ART types rank higher one particular Wilbur class – Scientific and Methodology, respectively. Hence, the mere ranking is not a good measure of which type performs best. Nevertheless, there is one aspect that makes a difference between them, also used to choose the best result: the standard deviation (SD) of the results between the Wilbur types. On average, all ART types score uniformly the Wilbur types, achieving an SD in the range (0–0.12]. However, some of them, see for example Observation (OBS), Hypothesis (HYP), Motivation (MOT) and Goal (GOA) in result set (I), have an increased SD (of around 0.25), which shows an increased discrimination between types – as we have previously anticipated. The ideal result would look like the one in result set (II), where in addition to the increased SD, all the other types have scored 0 across line. Unfortunately, such results are rare,and furthermore, they may lead to complete uniformity, e.g., results set (III), where all types have an increased SD and score almost in the same manner.

**Table 8:** Cross-corpus classification results.

		BAC	OBS	MET	CON	OBJ	RES	HYP	MOT	MOD	GOA
(I)	Generic	0.77	0.56	0.55	0.77	0.55	0.77	0.56	0.56	0.70	0.56
	Methodology	0.75	0.27	0.66	0.75	0.66	0.75	0.27	0.27	0.71	0.27
	Scientific	**0.90**	**0.74**	**0.69**	**0.90**	**0.69**	**0.90**	**0.74**	**0.74**	**0.84**	**0.74**
(II)	Generic	0.00	0.00	0.32	0.00	0.00	0.00	0.00	0.00	0.00	0.00
	Methodology	0.00	0.00	**0.82**	0.00	0.00	0.00	0.00	0.00	0.00	0.00
	Scientific	0.00	0.00	0.33	0.00	0.00	0.00	0.00	0.00	0.00	0.00
(III)	Generic	0.37	0.32	0.37	0.39	0.40	0.32	0.45	0.60	0.32	0.51
	Methodology	**0.83**	**0.82**	**0.83**	**0.84**	**0.84**	**0.82**	**0.86**	**0.92**	**0.82**	**0.90**
	Scientific	0.37	0.33	0.37	0.44	0.42	0.33	0.54	0.75	0.33	0.61

Cross-corpus classification using top 1% of the ART rules and a sequence-based serialisation. Bolded values denote the Wilbur types ranked highest by corresponding ART types.

## Discussion

The experiments described in the previous section led to a series of lessons learned, which are discussed below.

### Classification Results are to Some Extent Comparable to the Original Methods

At first glance, the classification results achieved in both corpora are fairly low, in particular for the ART corpus in the setting K = 1 (i.e., top 1). In practice, however, we observe that on average, the F1 score of the ART classification results for K = 3 is half the F1 score of the original method proposed by Liakata et al., while in the case of the Wilbur corpus, the results are better, the F1 being only 12% smaller for the Scientific type and 30% smaller for Methodology. The positive aspect of these results is that we have achieved them by relying, conceptually, on a single feature, the dependency structure of the sentences, as opposed to the mixture of linguistic and structural features – complemented by pre-processing steps – used by the original methods. From a different perspective, while our corpora analysis has shown that the rhetorical types seem to be fairly well defined, i.e., the overlap in rule sets is less than 50%, the classification results show that there is not enough discriminative power in the non-overlapping part of the rule sets. Furthermore, in the case of ART, the granularity of the rhetorical types appears to be too low to show a significant difference in the underlying style of the sentences structure.

**Figure 4:**
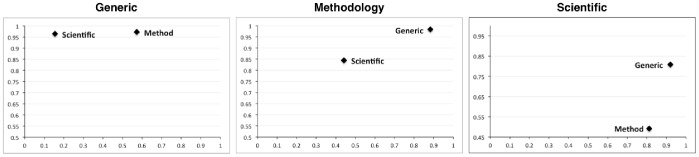
Pairwise sensitivity-specificity graphs for the Wilbur rhetorical types. The same type of analysis as depicted in Figure 3, but applied to the Wilbur corpus. The axes carry the same meaning as in Figure 3, i.e., the X axis denotes sensitivity, while the Y axis denotes specificity. In this case, the Generic type is shown to discriminate well against the Methodology type.

In order to get a better insight into the individual classification behaviour, we have compiled pair-wise confusion matrices for each rhetorical type in each corpus and computed the classifier's sensitivity and specificity. Fig. 3 depicts the confusion matrices computed on the best K = 1 classifier on the top 1% of the rules for the ART corpus. Here, we observe that most types can be associated with a particular cluster of types against which they discriminate well. This aspect can be used a deciding factor in an ensemble of classifiers that may initially restrain the set of possible classes using the discriminative preferences pointed by our approach, and then chooses the final class based on its underlying mechanisms. For example, Background (BAC) and Method (MET) and Result (RES) discriminate well against Motivation (MOT), Goal (GOA) and Hypothesis (HYP) – with a sensitivity over 90%, while Observation (OBS) and Model (MOD) discriminate well against Object (OBJ) – with a sensitivity over 75%. Among all types, the rule set of Motivation (MOT) has the lowest discriminative power, which is not particularly surprising, considering its lack of coherence – the set-based serialisation led to a rule set of over 500K rules from only 484 sentences. A similar behaviour is observable also in the Wilbur corpus, as depicted in Fig. 4– here, the measures have been computed on the best classifier on top 1% of the rules. Both the Scientific, as well as the Methodology types discriminate well against the Generic, however they have issues in discriminating against each other. In particular, the Scientific type has a low specificity with respect to Methodology, which leads to the conclusion that the rule set may be dominated by multiple patterns, some of which may be common to Methodology as well.

Based on the macro performance scores, we can conclude that, on its own, the dependency structure of the sentences is not enough to produce good classifiers for the complete set of rhetorical types. However, it has a very good applicability in building pair-wise classification mechanisms. This conclusion is also supported by the fact that, in the case of the ART corpus for example, the pairwise discrimination works well for difficult types. As shown in Fig. 3 and mentioned above, Background (BAC) and Method (MET) and Result (RES) discriminate well against types that achieved the lowest F1 scores in the automatic classification experiments performed by Liakata et al. [1], i.e., Motivation (MOT), Goal (GOA) and Hypothesis (HYP). Furthermore, these types have proven to be difficult also in the human annotation experiment discussed in the same article [1], by achieving an inter-annotator agreement, measured via Cohen's Kappa statistic, of less than 0.6– a value under the threshold of 0.7, which is being considered representative for inter-annotator agreement.

### There is No “One Size Fits All” Solution

As in any multi-class classification setting, and in particular in the cases with a large number of classes – e.g., ART, finding a classifier that performs well across all classes is challenging. This phenomenon is present also in our experiments. The different ranking mechanisms we have used filtered different sets of rules, which were able to exploit (or not) some of the patterns in the typed dependencies. Consequently, while the results we have presented in the individual classification experiments were not optimal across all rhetorical types, as a whole, the set of results was the best. In addition, there are two aspects that are worth remarking: (i) in the case of the ART corpus, we observe the major role played by the rules' *confidence* both in the initial filtering based on ranking – stand-alone, or part of the IR-Mixed setting – as well as in the final scoring; a similar role is carried out by *support* in the Wilbur corpus; and (ii) contrary to our expectations, in the ART corpus the set-based serialisation performed better than any of the sequence-based serialisations, with or without the *det* and *prep* dependency types.

Finally, with respect to the cross-corpus classification, the difference in granularity has clear implications on the classification, and hence, with a few exceptions, e.g., the Observation (OBS), Hypothesis (HYP), Motivation (MOT) and Goal (GOA) mapping to Scientific and the single Method (MET) to Methodology mapping, the results are unfortunately inconclusive.

### Less is More

This was a surprising finding of our experiments. Initially, we have expected the classification performance to be positively correlated with the number of rules used for classification. On the contrary, the results show that the efficiency is the same, or even lower, and that among the different sizes of the considered rule sets, the best classification can be achieved with top 1% of the rules, independently of the ranking mechanism. This finding is particularly important because it has a significant effect on the classification speed: using the top 1% the entire ART corpus can be classified in a few seconds, while increasing the rule set to the top 10% leads to an increasing in classification time of two orders of magnitude, i.e., it takes up to one hour.

### Common Typed Dependencies Don't Affect the Classification Efficiency

Finally, this last finding refers to the inclusion or exclusion of the often occurring typed dependencies in the rules. Our initial assumption was that *det* and *prep*, which have the highest support in both corpora (of over 91%) will act as “stop words” – i.e., will introduce noise, especially in the set-based serialisation. In reality, our experimental results have been completely agnostic to the presence or absence of these items.

## Conclusions

In this article, we have presented a study on using the dependency structure of sentences in performing corpus analysis and automatic recognition of conceptualisation zones. Our ultimate goal has been to provide a series of insights and lessons learned into the role carried by typed dependencies in defining patterns that describe the semantics of different rhetorical types. We have used an association rule mining process to generate rules from diverse serialisations of the dependency structure of sentences, followed by type-based classification according to different aggregation schemes.

Among other aspects, we have shown that given a reasonable granularity, e.g., similar to the one of the Wilbur scientific artefacts, it is enough to use a mere fraction of the emerging association rules to achieve good classification results. Our approach has limitations, especially in the context of a fine-grained multi-class classification. And while typed dependencies are not enough to produce complete classifiers (i.e., classifiers targeting the entire set of rhetorical types exposed, for example, by the CoreSC scheme), results show that they have a good applicability in deriving accurate pair-wise classifiers, in particular in the context of the most difficult rhetorical types.

## Supporting Information

Supporting Information S1 The experiments described in our manuscript have generated a substantial amount of data, which we cannot directly include in a document. Parts of this data are made available in the *supporting_information* archive, while the entire raw classification data is available on request. Below we briefly describe the content of this archive: **Table set S1.** **Basic rule set statistics on the ART corpus, including the sizes of the rule sets for each ranking mechanism we have used, for each rhetorical type at different thresholds: 1%, 5%, 10% and 15%. Table set S2. Basic rule set statistics on the Wilbur corpus, including the sizes of the rule sets for each ranking mechanism we have used, for each rhetorical type at different thresholds: 1%, 5%, 10% and 15%. Table set S3. Corpus-driven inter-type comparison on the ART corpus, looking at the overlaps between the rule sets of the different rhetorical types. Table set S4. Corpus-driven inter-type comparison on the Wilbur corpus. Table set S5. Corpus-driven intra-type comparison on the ART corpus, looking at the overlaps between the different serialization types in the context of a rhetorical type. Table set S6. Corpus-driven intra-type comparison on the Wilbur corpus. Table set S7. Cross-corpus type comparison, listing the overlaps in rule sets between the rhetorical types of the ART and Wilbur corpora.**
(ZIP)
